# Molecular Mechanisms in the Design of Novel Targeted Therapies for Neurodegenerative Diseases

**DOI:** 10.3390/cimb46060325

**Published:** 2024-05-29

**Authors:** Ilona Nowak, Marlena Paździor, Robert Sarna, Marcel Madej

**Affiliations:** 1 Silesia LabMed, Centre for Research and Implementation, Medical University of Silesia in Katowice, 18 Medykow Str., 40-752 Katowice, Poland; pazdzior.marlena@gmail.com (M.P.); robert90sarna@gmail.com (R.S.); mmarcel281297@gmail.com (M.M.); 2 Department of Molecular Biology, Faculty of Pharmaceutical Sciences in Sosnowiec, Medical University of Silesia, 40-055 Katowice, Poland

**Keywords:** molecular mechanisms, neurodegenerative diseases, central nervous system, multiple sclerosis, amyotrophic lateral sclerosis, Alexander disease, Huntington disease, spinal muscular atrophy

## Abstract

Neurodegenerative diseases are a diverse group of diseases characterized by a progressive loss of neurological function due to damage to nerve cells in the central nervous system. In recent years, there has been a worldwide increase in the expanding associated with increasing human life expectancy. Molecular mechanisms control many of the essential life processes of cells, such as replication, transcription, translation, protein synthesis and gene regulation. These are complex interactions that form the basis for understanding numerous processes in the organism and developing new diagnostic and therapeutic approaches. In the context of neurodegenerative diseases, molecular basis refers to changes at the molecular level that cause damage to or degeneration of nerve cells. These may include protein aggregates leading to pathological structures in brain cells, impaired protein transport in nerve cells, mitochondrial dysfunction, inflammatory processes or genetic mutations that impair nerve cell function. New medical therapies are based on these mechanisms and include gene therapies, reduction in inflammation and oxidative stress, and the use of miRNAs and regenerative medicine. The aim of this study was to bring together the current state of knowledge regarding selected neurodegenerative diseases, presenting the underlying molecular mechanisms involved, which could be potential targets for new forms of treatment.

## 1. Introduction

Neurodegenerative diseases are a heterogeneous group of diseases characterized by the progressive loss of neurological function due to neuronal damage in the central nervous system (CNS). Two types of nervous system disorders exist: acute and chronic. The former is characterized by a process in which neurons are rapidly damaged and typically die because of sudden trauma or traumatic events. Acute neuronal diseases include: traumatic brain injury, head injury, stroke, cerebral or subarachnoid hemorrhage and ischemic brain injury. Chronic diseases, on the other hand, such as Alzheimer’s disease (AD), Alexander disease (AxD), Huntington’s disease, Parkinson’s disease (PD), amyotrophic lateral sclerosis (ALS), and multiple sclerosis (MS), begin with a slow and progressive dysfunction of the nervous system, leading to cognitive, motor and/or sensory deterioration over time [1,2,3,4].

Chronic diseases are a major health problem. In recent years, their increased occurrence has been observed worldwide [5]. The frequency of neurodegenerative diseases is increasing as the population ages, and awareness and diagnoses of these diseases are also increasing. Approximately 70% of the population over the age of 65 suffers from a progressive neurodegenerative disease of the CNS, which is characterized by progressive memory loss, disorientation, language impairment, abnormal behavior and personality changes [3,6].

Despite intensive scientific research and clinical trials, no effective therapeutic agents have been developed [5]. The traditional management of neurological disorders only alleviates the severity of symptoms without eliminating the cause of the diseases, and the search for new treatments is hindered by the permeability of the blood–brain barrier (BBB) [7,8].

For these reasons, it is crucial to investigate the molecular mechanisms underlying this group of conditions. Firstly, this will allow us to identify the risk factors and pathophysiological mechanisms involved, which may lead to the development of new treatments. Secondly, better knowledge of the molecular mechanisms of these diseases may help identify biomarkers indicating disease progression, enabling earlier intervention and the monitoring of the course of these conditions.

Thus, the present review will outline the current state of knowledge regarding selected less frequently discussed and studied neurodegenerative diseases, such as MS, ALS, AxD, HD, and spinal muscular atrophy (SMA). The paper focuses on the molecular mechanisms involved in these diseases, along with future perspectives that may improve patient care and outcomes.

## 2. Amyotrophic Lateral Sclerosis and Multiple Sclerosis

### 2.1. Clinical Symptoms and Course of Amyotrophic Lateral Sclerosis and Multiple Sclerosis

Amyotrophic lateral sclerosis and multiple sclerosis both affect the nervous system and are classified as neurodegenerative diseases [9]. Both MS and ALS can lead to different and unpredictable disease progression. While periods of remission and the exacerbation of the disease are common in MS, in ALS, there is a gradual deterioration of muscle function [9,10,11].

Amyotrophic lateral sclerosis is difficult to diagnose in its early stages, and confusion with more common diseases is thought to delay the diagnosis of ALS [12]. Unlike MS, the initial symptom of ALS is muscle weakness, which progresses [10]. This occurs as a result of more or less extensive damage to peripheral and central nerve cells. The breakdown of this structure leads to a progressive neurodegenerative process that ultimately leads to the destruction of the nerve fibers supplying the muscles, and increased motor impairment leads to a gradual loss of the ability to perform basic and precise movements [10,13]. The complete loss of mobility and motor function adversely affects the muscles of the limbs and the function of other parts of the body, such as the respiratory system, esophagus and stomach. Consequently, this loss results in the progressive weakness of the muscles involved in limb movement, swallowing, speaking ad breathing [13].

On the other hand, MS is a chronic autoimmune disease of an inflammatory-demyelinating nature. One major change in MS is damage to the protective myelin sheath around the nerves in the brain and spinal cord due to inflammation, leaving demyelinated axons vulnerable to injury and degeneration [14]. In areas of myelin destruction, so-called sclerotic scars form, thus the origin of the name. In brain and spinal cord regions affected by multiple sclerosis, nerve transmission signals are reduced or blocked, resulting in neurological symptoms that may lead to reduced quality of life and disability. The occurrence and type of symptoms depend on the part of the CNS in which the myelin sheaths have been compromised [14,15].

### 2.2. Molecular Mechanisms of Amyotrophic Lateral Sclerosis and Multiple Sclerosis Pathophysiology

The molecular mechanisms underlying the pathophysiology of MS and ALS are still not completely understood [16]. Nevertheless, several major pathophysiological processes involved in ALS can be identified, resulting mainly from gain- or loss-of-function mutations in the approximately 40 known ALS genes. Only 10% of all ALS cases are familial (fALS), and these are closely linked to genes such as *SOD1*, *C9orf72*, *FUS*, or *TARDBP* [16,17]. However, the majority of cases, over 90%, are sporadic (sALS) and not directly associated with clearly defined, known genetic mutations [16,18]. The mechanisms behind sALS remain unclear, although research suggests that they may involve a number of factors acting together, rather than a single initiating event [16,19]. Genetic and phenotypic differences between fALS and sALS patients make it difficult to discover and draw conclusions about the pathogenic mechanisms behind the disease [19]. Some features and pathophysiological mechanisms exist that link these two ALS subtypes. The most common genetic cause is an expansion of hexanucleotide repeats in the *C9orf72* gene, which is responsible for 30–50% of familial ALS and 7% of sporadic ALS cases [20]. The TAF15 protein is mutated in both subtypes [21]. Mutations in the *TARDBP* and *FUS* genes, which encode the RNA-binding proteins TDP-43 and FUS, are responsible for 3–5% of fALS and <1% of sALS [20].

The overexpression of the gene encoding the wild-type Ewing’s sarcoma (EWS) RNA-binding protein (*EWSR1*) leads to neurodegeneration. The EWSR1 protein has been shown to have a diffuse distribution or dotted granular structure in patients with sALS [16,21]. In some forms of sALS, the cytoplasmic accumulation of TDP-43 is observed. Physiologically, TDP-43, is localized in the cell nucleus; in sALS, it begins to accumulate in the cytoplasm, forming protein inclusions. This phenomenon is an important part of ALS pathology, indicating potential abnormalities in RNA function and the regulation of gene expression, which may contribute to motor neuron degeneration [22].

**Figure 1:**
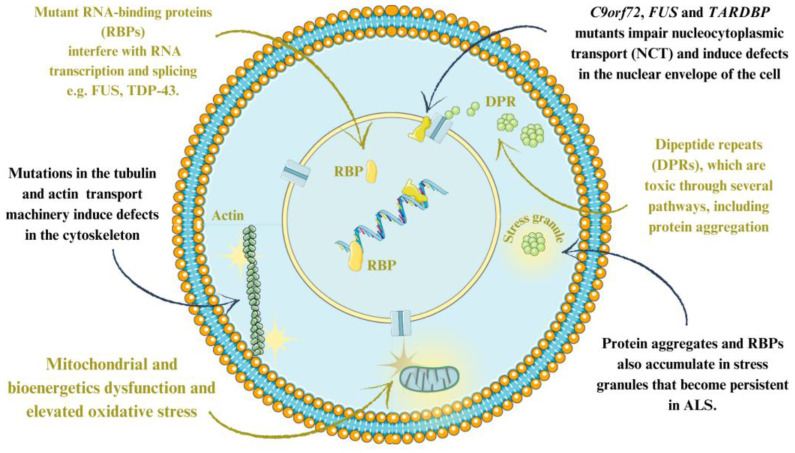
Pathological cellular processes in ALS. Mutations in ALS-associated genes lead to mutated RNA-binding proteins and damage to cellular and mitochondrial transport. Protein aggregation and mitochondrial mutations lead to energy disturbances and increased oxidative stress [13,16,23]. The figure was partly generated using Servier Medical Art, provided by Servier and licensed under a Creative Commons Attribution 4.0 unported license.

Four main categories of pathophysiological processes can be identified: impaired RNA metabolism, altered proteostasis/autophagy, cytoskeletal/transport defects and mitochondrial dysfunction (Figure 1) [13].

The formation of mutant RNA-binding proteins (RBPs; e.g., fused in sarcoma (FUS) and transactive response DNA binding protein 43 kDa (TDP-43)) interferes with RNA metabolism, resulting in extensive changes in transcription and splicing [13]. The C9orf72, FUS, and TARDBP mutants impair nucleocytoplasmic transport (NCT) and induce defects in the nuclear envelope of the cell [23]. The *C9orf72* gene is associated with frontotemporal dementia and/or ALS, with 50% of patients developing symptoms before the age of 58 years [24]. Dipeptide repeats (DPRs) are toxic due to the formation of protein aggregates, chromatin lesions and DNA damage [13].

In addition, the aggregation of cytoplasmic proteins (TDP-43 and superoxide dismutase (SOD1)) alters proteostasis and autophagy, preventing the removal of harmful proteins [13,25]. These processes are also blocked by mutations in vesicle-forming proteins (e.g., optineurin (OPTN), vesicle-associated membrane protein-associated protein B/C (VAPB), and valosin-containing protein). Protein deposits further block the endoplasmic-reticulum-associated protein degradation (ERAD) response and the ubiquitin proteasome system (UPS) [13,26].

Many mutated ALS-related genes, such as the tubulin (e.g., dynactin subunit 1 (*DCTN1*), kinesin family member 5A (*KIF5A*), and the tubulin alpha-4A chain) and actin (e.g., profilin 1) transport machinery, induce defects in the cytoskeleton or transport by inhibiting axon transport and the distribution of important organelles (e.g., the mitochondria) in cells [13,15].

Protein aggregates and mutations of mitochondrial protein components (e.g., coiled-coil–helix–coiled-coil–helix domain containing 10 (CHCHD10)) induce mitochondrial disfunction and bioenergetics and increase oxidative stress significantly [16]. Superoxide dismutase-induced mitochondrial dysfunction is a major feature of ALS. Impaired redox homeostasis in ALS due to the abnormal production of reactive oxygen species (ROS) and reactive nitrogen species (RNS) is also a molecular mechanism associated with multiple sclerosis [13,16,26].

The kinurenin pathway leads from projection-remitting multiple sclerosis (PRMS) to chronic progressive sclerosis [26].

The detection of a correlation between genetically determined ALS and the SOD1 mutation on chromosome 21 led to the hypothesis that the etiology of ALS may be closely linked to SOD1 overactivity [13,27,28]. Although mutations in the gene encoding SOD1 account for only 2% of all ALS cases, abnormal folded wild-type human SOD1 has been detected in both familial and sporadic ALS patients [27]. Lastres-Becker et al. [18] showed that sporadic and SOD1-ALS-immortalized lymphocytes indicate elevated levels of ROS and increased lipid peroxidation, mainly in sporadic ALS lymphoblasts, which is in accordance with the assumption that oxidative stress plays a key role in the pathogenesis of neurodegenerative diseases, including ALS [18]. The results support the hypothesis that an increase in the antioxidant pathway nuclear factor erythroid 2-relatd factor 2 (NRF2) and the inflammatory cytokines IL1-β and IL-6 can be observed in sALS lymphoblasts as a potential response to lipid peroxidation [18]. In addition, metabolomics studies allow for better insights into and diagnosis of MS. For diagnostic purposes, higher levels of scyllo-inositol and glutamine have been found in MS cases [26].

Multiple sclerosis is an autoimmune disease, and autoreactive immune cells play a key role in its pathophysiology [29]. Unknown antigen (AG), which is presented by major histocompatibility complex (MHC) class II molecules, stimulates the conversion of T cells to an autoreactive state, leading to inflammation [30]. The main antigen-presenting cells (APCs) are dendritic cells and macrophages (Mφ). Th presentation of AG by APCs activates T cells via MHC molecules. Na + concentrations can affect APC function, as well as matrix metalloproteinase (MMP) activity [30,31]. Autoreactive T cells diffuse into lymphoid tissues where they undergo proliferation [30]. Next, they enter the circulation after release by sphingosine-1-phosphate. Once stimulated, T cells attach to adhesion molecules, which are increased in expression, and begin to produce MMPs. The MMPs then cause a break in the BBB and attack the central nervous system (CNS). In the CNS, T cells interact with APCs and begin their proliferation. Myelin is attacked, and helper T cells develop into two types: pro-inflammatory T1 helper cells (Th1) and anti-inflammatory Th2 cells [31]. Th2 cells are responsible for releasing cytokines that attack Mφ cells and microglia, including tumor necrosis factor (TNF)-α and interleukin (IL)-1β, which may contribute to neurodegeneration through cytokine-induced cell death, the inhibition of glutamate reuptake in astrocytes, and the induction of dysfunctional ribonucleic acid [29,31]. In addition, T cells activate B cells to produce antibodies that cross the damaged BBB, leading to the formation of myelin autoantibodies [31]. Increased immunoglobulin levels in the CSF suggest a role one the part of B lymphocytes in multiple sclerosis [29,32].

In addition, B-lymphocyte antibodies initiate a complementary cascade that, again, attacks myelin [31]. Targeting inflammatory processes is the main goal of all current treatments for MS [31,33].

### 2.3. Potential Treatment for Amyotrophic Lateral Sclerosis and Multiple Sclerosis

Disease-modifying therapy is one of the most important treatments. There are few treatments for ALS that can slow the progression of the disease [10,11].

Currently, the FDA has approved three drugs for the treatment of ALS (Riluzole, Edaravone and Tofersen). Riluzole is thought to act by inhibiting the production of glutamate, a neurotransmitter that is theorized to initiate a series of molecular events that damage neurons. Edaravone is a radical scavenger, originally developed for the treatment of post-stroke patients [34,35,36]. Tofersen, an antisense oligonucleotide (ASO), binds to mRNA and reduces superoxide dismutase burden in patients with SOD1-ALS [15,16,23].

Several treatment options for MS are currently available [26,34,35]. Most cases respond to steroids (e.g., methylprednisolone) [34,35,36]. Multiple sclerosis biomarkers include increased or decreased levels of miRNAs that target several protective or altered pathogenic signaling pathways. Protective miR-199a, pathogenic miR-320, miR-155, miR-142-3p and miR-142 are increased in MS lesions or peripheral blood mononuclear cells (PBMCs) [12,37]. Other miRNAs, such as miR-219, miR-34a, miR-103, miR-182-5p, miR-124 and miR-15a/b, are downregulated in the cerebrospinal fluid (CSF) and PBMCs of patients with MS [12,37]. Low levels of miR-219 and high miR-150 in the CSF are new biomarkers that can help distinguish between MS and other neurological conditions. Another oxidative biomarker is nicotinamide adenine dinucleotide phosphate (NADPH) oxidase 2 (Nox2). This enzyme catalyzes the reduction of oxygen to produce ROS, which play an important role in the pathogenesis of MS. There is an urgent need for the development of ALS biomarkers to accelerate diagnosis, especially for atypical phenotypes, and enable better prognoses over the disease course [12,37].

## 3. Alexander Disease

### 3.1. Clinical Symptoms and Course of Alexander Diseases

Alexander disease (AxD) is a relatively rare neurodegenerative disease. It a type of leukodystrophy that typically manifests in infancy, although it can occur later in life [38,39].

Alexander diseases is a genetic disease that is difficult to differentiate from other white matter pathologies [38]. Typically located in the frontal lobes, lesions can occasionally result in heightened nervous system malfunction. Disease can be divided into two types depending on the location of the CNS lesions. Type one is characterized by early-onset convulsions, macrocephaly, progressive psychomotor and cognitive delays, and failure to thrive due to lesions in the forebrain [39]. Type two manifests as posterior brain lesions and associated symptoms such as palatal myoclonus, ataxia, dysphagia, and morphological dysfunction, and it can occur at any age [40,41]. The characteristic pathological features in both groups is the presence of Rosenthal fibers, which are aggregates of astrocyte cytoplasmic proteins, especially in perivascular, subcutaneous areas and subepithelial areas [42].

**Figure 2:**
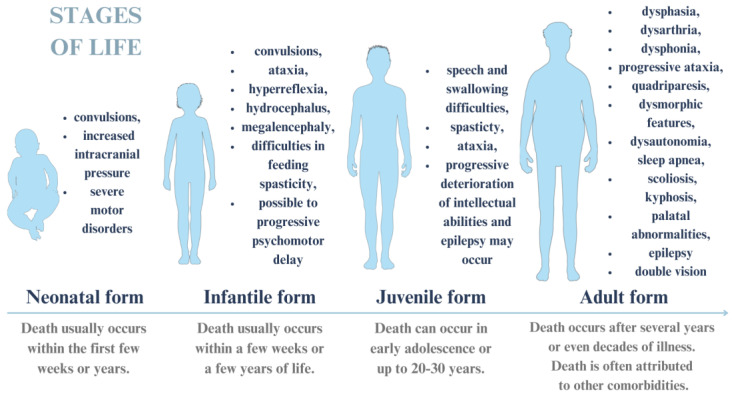
Alexander disease: symptoms occurring during a particular period of life. The figure was partly generated using Servier Medical Art, provided by Servier and licensed under a Creative Commons Attribution 4.0 unported license.

Symptoms of the disease vary depending on the age at which they occur (neonatal, infantile, juvenile, or adult), and may vary in severity as well. These symptoms are shown in Figure 2 [43,44].

### 3.2. Molecular Mechanisms behind Alexander Disease

Genetic mutations affecting the *GFAP* acidic filament protein cause Alexander disease. As of today, no recessive mutations have been reported among any of the *GFAP* mutations linked to the mentioned disease that have been found to be heterozygous [45]. The mutant proteins exhibit dominant behavior, disrupting the folding of the intermediate filament network and inducing the formation of characteristic protein aggregates, known as Rosenthal filaments, in the cytoplasm of astrocytes [46]. In addition to glial fibrillary acidic protein (GFAP), these fibers may also contain many other proteins that are involved in transcription (cJun), protein breakdown (ubiquitin and proteasome 20S), stress response (αB-crystallin and Hsp27), and other processes (vimentin, plectin, and synemin) [47,48,49]. Neurodegeneration results from intercellular communication with neurons and potentially with the oligodendrocytes generating the myelin surrounding axons due to the degradation of auxiliary activities and the toxic effects of astrocytes expressing mutant GFAP [48,49]. Glial fibrillary acidic protein inhibits polymerization and may cause the creation of aberrant oligomers. Increased GFAP in AxD is likely caused by increased transcription and protein synthesis linked to the astrocyte stress response, while the exact mechanisms behind GFAP accumulation and aggregation are unclear [50,51]. The CNS expresses many variations of the GFAP transcript. There are various types of Alexander disease caused by distinct *GFAP* mutations [52]. Nearly 100% of *GFAP* mutations are de novo and have high penetrance. About 90% of the transcripts are made up of the dominant GFAPα isoform, 4% are made up of GFAPδ (also known as ε), and the remaining transcripts are composed of GFAPκ and λ at lower levels [51].

### 3.3. Potential Treatment for Alexander Disease

Clinically, given the nonexistence of treatment, the helpful approaches utilized thus far have been based on treating symptoms. These include antiepileptic drugs (e.g., carbamazepine (CBZ) and phenytoin (PHT)); supportive strategies and surgical procedures such as valve surgery for hydrocephalus [53]. Taking all this into account, there is no standard treatment for AxD. Despite of the various models created over the past two decades, only modest improvements have been made in identifying an essential role on the part of astrocytic clutter in leukodystrophy [53]. It is still unclear whether white matter shortages result from hypo- or demyelination and whether this depends on patient age and the area of infection. Nevertheless, recent studies have made noteworthy progress in this area [54]. Because anomalous collapsing may be a common trademark of mutant and over-the-top GFAP protein, most of the defensive techniques examined to date have endeavored to lower GFAP levels by focusing on its expression. Among the compounds tried, phenytoin (5,5-diphenylhydantoin), carbamazepine (a derivative of iminostilbene with a dibenzazepine nucleus; thus, it has a tricyclic structure similar to clomipramine), and curcumin were utilized in AxD cell models to normalize *GFAP* expression levels [55]. It enhanced fiber coiling and organization, while ceftriaxone decreased expression and activated end of mutant *GFAP* [39,55]. Pexidartinib (a colony-stimulating factor 1 receptor (CSF1R) inhibitor) increased GFAP levels and decreased macrophage numbers in a mouse model of AxD [55]. Importantly, antisense oligonucleotides that reduce *GFAP* expression have shown beneficial effects in a recently developed rat model that appears superior to mouse models and has helped in designing the first clinical trial with AxD patients [51,56]. Targeted ASO therapy offers GFAP suppression as a viable treatment option for AxD. In fact, GFAP-targeting ASO proof-of-concept experiments in mouse models have shown that AxD pathology, including protein aggregation, accumulation, and the reactive stress response, can be reversed [57]. However, improvement in animal models with clinically meaningful outcome measures is required because AxD mice exhibit significant behavioral abnormalities [56,58]. With high levels of GFAP, myelin deficiencies, and functional impairments, including motor impairment, failure to thrive, and increased mortality, the GFAP-R237H rat carries a significant pathological burden [56]. The severe and progressive phenotype of the R237H rat has led to the investigation of two treatment approaches to preventing common behavioral abnormalities [56]. Specifically, GFAP-targeting ASO treatment for R237H rats at weaning eliminated molecular and cellular damage, delaying the emergence of clinical phenotypes [56]. The treatment of severely afflicted young adult rats resulted in a significant improvement in total neurologic function, partially reversing motor impairment and white matter impairments, in addition to eliminating pathology [56,59].

## 4. Huntington’s Disease

### 4.1. Clinical Symptoms and Course of Huntington’s Disease

Huntington’s disease is an autosomal dominant genetic disorder, and is a neurodegenerative disease [60,61,62,63]. The incidence of this disease varies greatly and depends on ethnicity [60,61]. Incidence ranges from 0.01 to 13.7 per 100,000. Huntington’s disease is most common in the Caucasian population, while HD is rarely seen in Asian and African countries [61]. The main symptoms of HD include movement disorders, neuropsychiatric symptoms, neuropsychiatric symptoms, cognitive impairment, weight loss and diurnal cycle disorders [62]. Currently, HD is monitored using the Huntington’s Disease Rating Scale (UHDRS). The UHDRS is based on the determination of motor, cognitive, and behavioral abnormalities and functional capacity, which is accomplished by observing clinical symptoms [63]. Due to the subjective nature of determining disease progression, it is important to develop specific markers to help determine the progression of the disease [60,63]. Currently, no effective treatment for HD is available. Existing therapies focus on treating the main disease via vesicular monoamine transporter type 2 (VMAT2) inhibitors, antipsychotics, *N*-methyl-D-aspartate (NMDA) receptor antagonists and autophagy inducers [61].

### 4.2. Molecular Mechanisms of Huntington’s Disease

Huntington’s disease is caused by a mutation in the huntingtin gene (HTT), at *loci* 4p16.3. The disease is initiated by an expansion of three-nucleotide CAG repeats in the region of exon 1 of HTT encoding polyglutamine [61]. The length of the repeat encoding polyglutamine in HTT exon 1 shows polymorphism within ethnic groups, resulting in a differential frequency of HD [61,64]. It has been observed that the number of HTT CAG alleles influences age at disease manifestation. Classes have therefore been constructed to define the penetration of HD depending on the number of alleles: high normal (>26; <36 repeats CAG), reduced HD penetration (>35; <40 repeats CAG) and full HD penetration (>39 repeats CAG). It has been observed that with an increase in the number of CAG repeats, HD decreases age at disease manifestation [60,64]. Although, due to this phenomenon, it is possible to predict whether HD will develop, there is a lack of knowledge about how to determine the exact age at disease manifestation [60,64].

The mutant HTT (mHTT) protein affects the impairment of a number of cellular processes, which ultimately contributes to neuronal cell death [65]. Among the cells most sensitive to mHTT are medium-sized striatal spiny neurons, although the death of cortical neurons has also been observed [65]. Repeats CAG in mHTT affect its post-translational modification affecting its stability, subcellular distribution, cleavage, and function [65]. The mutated HTT protein also influences the correctness of transcription, which leads to increased immune response, mRNA processing or even decreased metabolic processes and synaptic function impairment [65]. Moreover, the ability of mHTT to migrate between cells has been indicated [65].

The mechanisms contributing to the development of HD also include mitochondrial dysfunction [65,66,67,68]. In HD, mitochondrial energy production is compromised, oxidative stress is increased, and mitochondrial quality control and distribution are affected. mHTT also disrupts mitochondrial calcium metabolism and promotes mitochondrial-dependent cell death [66,68]. Interestingly, Liu et al. [67] indicate the ability of mHTT to disrupt the heat shock response by affecting the function of heat shock transcription factor 1 (HSF1), leading to mitochondrial dysfunction [67,68].

### 4.3. Potential Treatment for Huntington’s Disease

Antisense oligonucleotides are short, synthetic, single-stranded DNA molecules of approximately 18–30 nucleotides in length [65,69]. They can degrade mRNA via an endonuclease (RNase) [65,69]. Molecules of ASO not only bind to mRNA to degrade it, but also prevent the formation of a 5′cap, thereby interfering with the splicing process [64]. Antisense oligonucleotides target introns and exons; their action is reversible; and, importantly, they do not saturate endogenous pathways [65,66]. These small DNA molecules also have the ability to penetrate the CNS, and they are easily captured by glial and neuronal cells [65]. The use of ASO in an HD mouse model significantly reduced the amount of mHTT, which led to an improvement in the mice’s neurological function. One example of the use of ASO in practice is an antisense oligonucleotide designed to inhibit HTT (IONIS-HTTRx), which in transgenic mice with human mHTT reduced its expression by as much as 80% and reduced the amount of mutant protein by two-thirds [69]. The effect after IONIS-HTTRx was observed for 4 months. After treatment, a significant improvement in motor function was observed, with some individuals almost completely regaining motor function [69]. Following the administration of IONIS-HTTRx in transgenic mice, a nearly 50% reduction in mHTT in the cortex and a 15–20% reduction in the mHTT of the striatum was observed. In a human study, a significant decrease in the concentration of mHTT in CSF was observed [69].

Ribonucleic acid interference (RNAi) is an endogenous cellular process that occurs via miRNAs. During this process, hairpin miRNAs form an interfering complex that prevents translation of the target mRNA [69]. The translation process is also interfered with by siRNAs, which show similar effects to miRNAs but do not form hairpin structures and are derived from longer double-stranded RNAs and show greater specificity to the target [65]. Molecules such as siRNAs or miRNAs must, however, be delivered by packaging them in carriers. Carriers for siRNA/miRNA can be liposomes, recombinant viruses or nanoparticles [65]. Caron et al. [70] used a recombinant adeno-associated virus (AAV5) viral vector to deliver miRNA to the brain striatum of a humanized HD Hu128/21 mouse model to reduce mHTT levels. The viral vectors loaded with miRNAs transduced only neuronal cells, leading to reduced mHTT levels and improved behavioral and neuropathological symptoms [70]. It was concluded that the decrease was associated with impaired motor coordination and striatal atrophy. Therefore, further research is needed on the use of a research model using wild-type HTT, as well as the potential silencing of mHTT in the body [70].

### 4.4. CRISPR-Cas9 in Huntington Disease Therapy

The clustered regularly interspaced short palindromic repeats-associated protein 9(CRISPR-Cas9)-genome-editing method has been successfully applied in biomedical research [71]. The system consists of two key components: Cas9 nuclease and single-guide RNA (sgRNA). The sgRNA determines the specificity of the target DNA sequence based on integrity to specific fragments [71]. Once the target sequence is recognized, the Cas9 nuclease causes a double-stranded break, which is then repaired by error-prone non-homologous end joining, which can lead to pathology due to a shift in the reading frame; this, in turn, results in impaired gene expression [71]. Importantly, the CRISPR-Cas9 system allows the use of multiple sgRNAs. Using this system, it is also possible to edit the genome in terms of mHTT [71]. CRISPR-Cas9 has been successfully applied to cell and animal lines, in which CAG repeats in exon 1 of HTT were excised. The modification performed resulted in reduced mHTT levels, astrocyte reactivity, and improved motor function [72]. Now, an improved version of the CRISPR-Cas9 edition has been developed, in which it is possible to cut out and replace the excised fragment with the corrected fragment, without the need to create double-strand breaks using Cas9 nickases and pegRNAs, which are modified components of the original system [72]. The overall system enables a permanent reduction in the expression of the mHTT allele and reduces the risk of pathogenic consequences because of using the system. Advances in editing CRISPR-Cas9 genes show that in the future, it may be successfully used in limiting mHTT expression in HD [73]. Recombinant viral vectors have been successfully used to test for neurodegenerative diseases, such as HD, Canavan disease, Parkinson’s disease, MS, or glioblastoma multiforme [8]. Importantly, the use of such vectors may be necessary when using siRNA, miRNA or CRISPR-Cas9, which are currently showing great potential in terms of creating therapies for HD [8,69,70,71,72,73]. Several gene therapy strategies for the treatment of Huntington’s disease are presented in Table 1.

## 5. Spinal Muscular Atrophy

### 5.1. Molecular Pathogenesis of Spinal Muscular Atrophy

Spinal muscular atrophy (SMA) is an autosomal neuromuscular disease primarily caused by a mutation in the survival motor neuron-1 (*SMN1*) gene, which is located on chromosome 5q13 and responsible for the proper survival of motor neurons [82,83,84]. The malfunction of SMN protein leads to the degradation of alpha motor neurons in the anterior horns of the spinal cord, thereby resulting in movement-related defects (i.e., progressive sympathetic weakness of the proximal muscles, speech problems, paralysis and eventually respiratory muscle weakness or atrophy), depending on the phenotype of the disease [82,83,84]. The molecular mechanisms of SMA are mainly related to copy number abnormalities in the *SMN1* and *SMN2* genes. The telomeric region usually contains only one copy of *SMN1*, while the centromeric region contains several copies of *SMN2* [85,86]. The main difference between such genes is due to variations caused by the nucleotide sequence within eight nucleotides. In most cases of SMA disease, mutations based on a deletion of the *SMN1* gene are present, leading to a complete loss of expression for this gene and, therefore, a deficiency in the formation of a functional protein [85,86]. The second significant gene in terms of the disease phenotype is *SMN2*, the variable copy number of which determines the severity of SMA [85,86].

**Figure 3:**
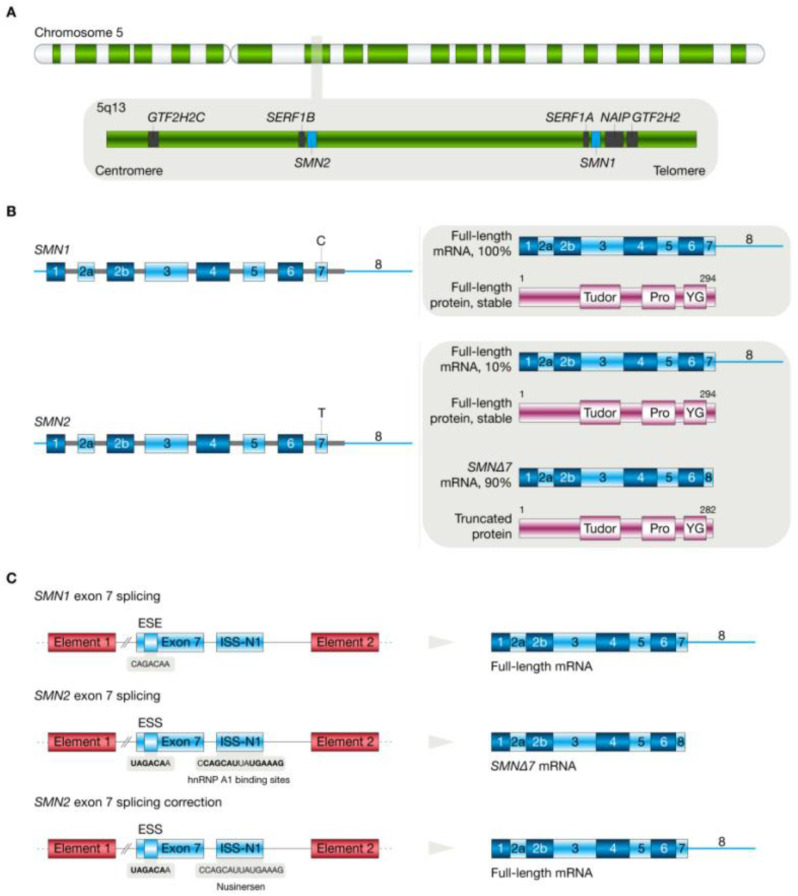
Molecular mechanisms of spinal muscular atrophy development. Wild-type chromosome 5q13 is covered by the telomeric *SMN1* gene and the centromeric *SMN2* gene (**A**); splicing pre-mRNA *SMN1* produces full-length mRNA (**B**); exon 7 splicing in *SMN1* and *SMN2* genes (**C**). Reprinted with permission from Ref. [87]. Copyright 2022 MDPI.

The molecular background of the production of a defective form of SMN protein is a disturbance in the alternate splicing of *SMN2*, which results in the production of an isoform of mRNA that has been shortened by one nucleotide (the absence of exon 7) [87,88,89]. It is assumed that the silent conversion of cytosine to thymine at position 6 of this exon is crucial in the process, leading to the suppression of the exonic splicing enhancer element (ESE). This, in turn, results in the formation of the exonic splicing silencing element (ESS), to which the A1/A2 splicing factor can bind [87,89]. This leads to the translation of the transcript into a dysfunctional SMNΔ7 protein, which is subsequently degraded via a proteasome-associated pathway [90,91,92,93]. Accordingly, due to this molecular mechanism, current applied therapies mainly consist of the modification of the splicing phase or the correction of the *SMN2* gene in the exon 7 region [87,88,89]. The molecular mechanism of SMA is shown in Figure 3.

### 5.2. Clinical Symptoms and Course of Spinal Muscular Atrophy

The classification system for SMA, which is mainly based on the number of copies of the *SMN2* gene, consists of types 0-IV, with a range of clinical manifestations and survival times since detection [90,91]. Type 0 SMA, in the course of which only one copy of *SMN2* is present, mainly affects the prenatal period, while after birth, children are characterized by severe hypotonia and respiratory failure, leading to death within 6 months [90,91].

The presence of two copies of the *SMN2* gene indicates type I SMA, which is also called Werdnig–Hoffmann disease. It is the most common type of SMA, reaching about 50–60% of cases [90,91]. The clinical features include difficulty swallowing and sucking, as well as hypotonia and difficulty holding the head [87,90,91]. It mainly affects children under 6 months of age [90]. Two or three copies of the *SMN2* gene are characteristic of SMA type II (Dubowitz disease). It affects mainly children over 6 months of age, manifesting in the form of hand tremors, progressive proximal muscle weakness, scoliosis, or lack of deep tension [87,90,91]. Type III SMA (Kugelberg-Welander disease) is typical among children older than 18 months, and their copy number of the *SMN2* gene is typically four [87,91]. In this case, patients reach adulthood, and difficulties with standing or climbing stairs are observed as part of the clinical pattern. Lastly, type IV SMA refers to adults [90,91]. The clinical features are relatively non-specific: however, abnormal muscle weakness is observed, as well as increased susceptibility to fatigue. In this case, the *SMN2* copy number can be above four [90,91].

### 5.3. Potential Treatments for Spinal Muscular Atrophy

Currently, the main therapies for SMA are based on the use of antisense oligonucleotides targeting the *SMN2* exon 7 alternate splicing mechanism in adult patients [87,88]. In contrast, in children, an approach based on the correction of the *SMN1* gene using AAV-based vector therapy is preferred [87,88]. The first approach utilized Food and Drug Administration (FDA)-approved nusinersen. The mechanism of action of the drug is based on targeting the intron splicing silencing sites (ISS-N1) of the *SMN2* gene, so that exon 7 is not excised, thereby increasing protein production [87,88]. The drug is administered to patients intrathecally via lumbar puncture. A similar mechanism of action is observed with the second drug approved by the FDA, risdiplam, which, in contrast to nusinersen, is administered orally [87].

Regarding targeting the *SMN1* gene, on the other hand, the only drug approved to date is onasemnogene abeparvovec [87,92]. The viral carrier used in this instance is a recombinant adeno-associated serotype 9 virus (AAV9) that carries a complementary DNA (cDNA) transgene of the *SMN1* gene, which encodes a fully functional protein [87,91,92,93]. The advantage of this system is the fact that the viral vector targets the motor neuron cell. It is administered in the form of suspension via intravascular infusions [91,92,93].

Considering the fact that more and more cases of SMA are being diagnosed each year, clinical trials are also currently being conducted on new therapeutic substances that can potentially be used in the treatment of SMA.

### 5.4. Novel Treatment Options for Spinal Muscular Atrophy

Spinal muscular atrophy may manifest at any stage of life, from infancy to adulthood [92]. Despite relatively good diagnostics focusing on molecular testing using polymerase chain reaction (PCR)-based methods and methods based on short-read sequencing, treatment options are mainly limited to the three FDA-approved pharmaceuticals [92,93]. Due to this, new solutions in the field of genetic engineering and pharmaceutics are being sought to develop alternative methods of treating SMA with the highest possible efficacy.

In a study conducted by Theil et al. [94] on a neonatal and juvenile transgenic mouse model with an exosome 7 deletion in the *SMN2* gene (SMNΔ7), as well as Wistar Hannover rats and beagle dogs, the researchers evaluated the efficacy of branaplam [94]. Branaplam is used to correct mRNA splicing, and its mechanism involves the increased binding affinity of the small nuclear ribonucleic protein U1 (snRNP) to the *SMN2* splicing site [94]. The drug has been administered daily using oral gavage for a specific period of time, depending on the model [94]. For transgenic mice, this was for 33 days (0.1 and 3 mg/kg), for Wistar rats from 13 to 26 weeks at a dose of 2 mg/kg, and for dogs for a period of 13 weeks at a dose of 2 mg/kg [94]. Following this period, tissue sections of the cerebellum, striatum and dentate gyrus were analyzed using immunohistochemical methods, and the pharmacokinetic profile was evaluated [94]. The results showed that the patterns of proliferation, migration, and neuronal innervation in the studied areas did not differ after drug administration as compared to controls [94]. The researchers concluded that orally administered branaplam does not affect neurogenesis among young animals, which may also be reflected in young SMA patients; however, this requires further research at the clinical stage [94].

A relatively new approach was taken by Ottesen et al. [95], who studied the synergistic effect of using ASO anti-N1 in combination with risdiplam and branaplam. For this purpose, they used primary fibroblast cells isolated from SMA patients (line GM03813), who were exposed to single compounds and their combinations [95]. Using reverse transcription PCR (RT-PCR) and RNA-seq, they characterized and compared the transcripts. They concluded that the use of combination therapy has a negligible effect on changing the gene expression profile if it is administered at low dosages [95]. However, further studies in an animal model are required, along with an increase in targeted therapies [95]. A similar study was also conducted by the team of Ishigami et al. [96] on the effects of the synergistic action of risdiplam and branaplam [96].

Undoubtedly, most of the studies conducted using animal models are followed by clinical trials evaluating the effectiveness of the therapy and its efficiency in patients in different age groups. Clinical trials using small molecules to treat SMA in different age groups of patients are currently ongoing. Some of them are presented in Table 2. These were selected from the following database: https://clinicaltrials.gov (accessed on 27 March 2024).

The limited methods for treating SMA have prompted the search for new therapeutic approaches, which are mainly aimed at restoring a fully functional SMN protein with a high efficacy and long-lasting effect. Thus, increasingly new formulations and gene-engineering technologies are being developed to generate viral carriers for targeted therapy and exosomes to act as nanocarriers [97,98]. It may also be an interesting approach to use detected abnormalities in the expression of the genes responsible for the phenotypic symptoms of the disease as biomarkers of SMA, which may vary from stage to stage, as in the case of glioblastoma multiforme [97,99].

## 6. Future Perspectives

The above-mentioned molecular strategies, such as ASO, si/miRNA, and CRISPR-Cas9, are beginning to be successfully applied to ALS, AxD, HD, and SMA therapies. A prime example of the use of the knowledge of the molecular mechanisms of disease in designing therapies is the ASO molecules used in the treatment of ALS (targeting and silencing the expression of mutant *SOD1*), AxD (silencing the expression of mutant *GFAP*), and HD (reducing the expression of *mHTT*) [27,28,45,65]. Knowledge of the precise molecular mechanisms behind ALS, AxD, HD, and SMA may reveal new potential molecular target.

The CRISPR-Cas9 gene-editing system is also noteworthy because it is being extensively tested for use in editing defective genes, which may lead to the correction of mutated molecules in the cell and thus, an improved phenotype. The reduction in mHTT levels in cell lines and animals after CRISPR-Cas9 treatment is an example of such action. Moreover, the first drug based on the CRISPR-Cas9 gene-editing system (CTX001™), which has been FDA-approved for the treatment of transfusion-dependent beta thalassemia, is already emerging [100]. Thus, this stream of research seems to bring new perspectives to personalized therapy for the described diseases.

Strategies for ASO, si/miRNA, and CRISPR-Cas9 delivery based on AAV or nanoparticles, improving their penetration, specificity, and stability, are also discussed in the paper. In addition, due to their small size, they can overcome barriers (i.e., the BBB) which makes them ideal for CNS disease therapy.

Considering the aggregation of proteins in ALS (TDP-43 and SOD1 aggregation), AxD (mutant GFAP aggregation), and HD (mHTT aggregation) and mutations in key genes for the above-mentioned diseases, combining ASO, si/miRNA-mediated gene silencing therapy, and the CRISPR-Cas9 gene editing system may be a solution. Moreover, due to similar delivery strategies for these therapeutic molecules, it may be possible to combine them in a single vehicle or delivery strategy (AAV, infusions, or injections). Interesting results can be obtained by combining known therapies with therapies involving ASO, si/miRNA and CRISPR-Cas9 engineering.

However, the limitations of the therapies should not be overlooked, which may be the key to modifying them. Among the most significant limitations are the distribution (intrathecal administration and intravenous injection) and stability of the molecules affecting molecular pathways and the multiplicity of modulated functions. In these respects, the focus should be on developing new drug formulations to reduce the invasiveness of drug delivery.

## 7. Conclusions

This review presents numerous studies on neurodegenerative diseases, their molecular mechanisms, and the use of knowledge about them to develop new treatments and diagnostics. The diseases listed have mainly symptomatic treatments that alleviate the course of the disease in question, improving patients’ quality of life.

Promising results are being shown in studies on reducing GFAP levels in Alexander disease, which may lead to the first clinical trials. Additionally, the use of ASO in a mouse model of Huntington’s disease significantly reduced the mutant HTT (mHTT) protein, resulting in improved neurological function. Advances in CRISPR-Cas9 gene editing indicate that it can be effectively used in the future to reduce mHTT expression in Huntington’s disease. The data presented in the following paper indicate that the use of specific biomarkers, as in the case of MS, allows for the rapid and accurate diagnosis of diseases, enabling the faster initiation of treatment and the avoidance of complications. The personalization of therapy based on biomarkers improves the effectiveness of treatment, so it is essential that specific biomarkers are sought for ALS, SMA, and other neurodegenerative diseases.

Nevertheless, to strive for the most effective therapies and diagnostics, further research into new and known molecular mechanisms is needed to better understand how they work, their strengths and weaknesses, and opportunities to improve current methods.

## Figures and Tables

**Table 1:** Selected examples of studies using ASO, si/miRNA, CRISPR-Cas9 in Huntington disease.

Type of Therapy	Therapeutic	Model Organism	Delivery Strategy	Therapeutic Effect	Ref.
miRNA	AAV5-miHTT	Mice R6/2 HD	Bilateral AAV5-miHTT injection into the thalamus, intradurally	Improved motor coordination, increased survival time, alleviation of neuronal dysfunction, reduced changes in striatum and cortex	[74]
miRNA	AAV5-miHTT	Mice Q175 HD	Injection of AAV5-miHTT into the striatum	Dose-dependent reduction in mHTT aggregates in striatum and cortex, improved dyskinesia, prolonged survival time media	[74]
miRNA	AMT-130	Patients HD	Injection of AAV5-miHTT into the striatum	Improved motor coordination and long-term reduction in mHTT, high specificity and no off-target effects observed	[75,76]
siRNA	cc-siRNA-Htt	Mice	Injection into the right striatum	Weakening of motor abnormalities and reduction in mHtt expression	[77,78]
ASO	MkHuASO	Rhesus monkeys	Administration of Aso into the cerebrospinal fluid	Sustained reduction in Huntington mRNA in most areas of the brain and spinal cord	[78,79]
CRISPR-Cas9	dCas9-sgRNA	Mice R6/2 HD	Injection into the striatum	Improves motor functions and delays their progression	[80]
CRISPR-Cas9	SpCas9-NG	Mice R6/2 HD	Microinjection into the mouse zygote	Contraction of CAG repeats, improvement of HD phenotype	[81]

miRNA—microRNA; AAV5-miHTT—engineered miRNA adeno-associated virus vector 5; HD—Huntington disease; AMT-130—miRNA introduced into the target cells using adeno-associated virus (AAV) of serogroup 5; mHTT—mutant huntingtin; siRNA—small interference RNA; cc-siRNA-Htt—cholesterol-conjugated small interfering RNA duplexes (cc-siRNA) targeting human Htt mRNA (siRNA-Htt); ASO—antisense oligonucleotide; MkHuASO—ASO completely complementary to both Rhesus monkey and human huntingtin mRNA; CRISPR-Cas9—clustered regularly interspaced short palindromic repeats associated protein 9; dCas9-sgRNA—dead Cas9 single guide RNA; SpCas9-NG—Streptococcus pyogenes Cas9 recognizing NGG protospacer adjacent motif.

**Table 2:** Clinical trials for the treatment of spinal muscular atrophy found at https://clinicaltrials.gov (accessed on 27 March 2024).

Research Title	Drug/Molecule	Status	Application Route	NCT Number	Phase	Participants
A Study to Evaluate Higher Dose (HD) Nusinersen (BIIB058) in Participants with Spinal Muscular Atrophy Previously Treated with Risdiplam (ASCEND)	Higher Dose Nusinersen (BIIB058)	Recruiting	Intrathecally	NCT05067790	III	45
Study of the Functional Effects of Nusinersen in 5q-Spinal Muscular Amyotrophy Adults (SMA Type 2 or 3 Forms) (NUSI-AD-5qSM)	Nusinersen	Recruiting	Intrathecally	NCT04576494	N/A	24
Clinical Trial to Assess the Safety and Efficacy of EXG001-307 in Patients with Spinal Muscular Atrophy Type 1	EXG001-307	Recruiting	Intravenously injection	NCT05614531	I/II	12
A Clinical Study Evaluating the Safety and Efficacy of SKG0201 Injection in Patients with Spinal Muscular Atrophy Type 1	SKG0201	Recruiting	Injection	NCT06191354	N/A	12
Evaluation of Safety and Efficacy of Gene Therapy Drug in the Treatment of Spinal Muscular Atrophy (SMA) Type 1 Patients	GC101	Recruiting	Intrathecally	NCT05824169	I/II	18
Phase IIIb, Open-label, Multicenter Study to Evaluate Safety, Tolerability and Efficacy of OAV101 Administered Intrathecally to Participants with SMA Who Discontinued Treatment with Nusinersen or Risdiplam (STRENGTH)	OAV101	Active, non-recruiting	Intrathecally	NCT05386680	IIIb	27

N/A—not applicable.

## Data Availability

Available on request and with regulations.
